# Tissue biodistribution of intravenously administrated titanium dioxide nanoparticles revealed blood-brain barrier clearance and brain inflammation in rat

**DOI:** 10.1186/s12989-015-0102-8

**Published:** 2015-09-04

**Authors:** Clémence Disdier, Jérôme Devoy, Anne Cosnefroy, Monique Chalansonnet, Nathalie Herlin-Boime, Emilie Brun, Amie Lund, Aloïse Mabondzo

**Affiliations:** CEA, Direction des Sciences du Vivant, iBiTec-S, Service de Pharmacologie et d’Immunoanalyse, Equipe Pharmacologie Neurovasculaire, 91191 Gif-sur-Yvette, France; INRS, Département Polluants et Santé, Rue du Morvan, CS 60027, 54519 Vandœuvre Cedex, France; DSM, IRAMIS, NIMBE (UMR 3685), laboratory of Nanometric Structures, CEA Saclay, 91191 Gif/Yvette, France; Laboratoire de Chimie Physique, UMR CNRS 8000, Université de Paris-Sud, 91405 Orsay, France; Department of Biological Sciences, University of North Texas, Denton, TX USA

## Abstract

**Background:**

Notwithstanding increasing knowledge of titanium dioxide nanoparticles (TiO_2_ NPs) passing through biological barriers, their biodistribution to the central nervous system (CNS) and potential effects on blood-brain barrier (BBB) physiology remain poorly characterized.

**Methods:**

Here, we report time-related responses from single-dose intravenous (IV) administration of 1 mg/kg TiO_2_ NPs to rats, with particular emphasis on titanium (Ti) quantification in the brain. Ti content in tissues was analyzed using inductively coupled plasma mass spectrometry. Integrity and functionality of the BBB as well as brain inflammation were characterized using a panel of methods including RT-PCR, immuno-histo chemistry and transporter activity evaluation.

**Results:**

Biokinetic analysis revealed Ti biopersistence in liver, lungs and spleen up to one year after TiO_2_ NPs administration. A significant increase of Ti in the brain was observed at early end points followed by a subsequent decrease. In-depth analysis of Ti in the total brain demonstrated quantitative Ti uptake and clearance by brain microvasculature endothelial cells (BECs) with minimal translocation in the brain parenchyma. The presence of Ti in the BECs did not affect BBB integrity, despite rapid reversible modulation of breast cancer resistance protein activity. Ti biopersistence in organs such as liver was associated with significant increases of tight junction proteins (claudin-5 and occludin), interleukin 1β (IL-1β), chemokine ligand 1 (CXCL1) and γ inducible protein-10 (IP-10/CXCL10) in BECs and also increased levels of IL-1β in brain parenchyma despite lack of evidence of Ti in the brain. These findings mentioned suggest potential effect of Ti present at a distance from the brain possibly *via* mediators transported by blood. Exposure of an *in vitro* BBB model to sera from TiO_2_ NPs-treated animals confirmed the tightness of the BBB and inflammatory responses.

**Conclusion:**

Overall, these findings suggest the clearance of TiO_2_ NPs at the BBB with persistent brain inflammation and underscore the role of Ti biopersistence in organs that can exert indirect effects on the CNS dependent on circulating factors.

**Electronic supplementary material:**

The online version of this article (doi:10.1186/s12989-015-0102-8) contains supplementary material, which is available to authorized users.

## Background

Titanium dioxide (TiO_2_) nanoparticles (NPs) are the second most frequently used NPs in industry worldwide (http://www.nanotechproject.org). TiO_2_ is widely used as a white pigment in paint, ink, plastic, and paper and as food additive, while the nanosized TiO_2_ is also used for its photocatalytic activity in self-cleaning materials and for its UV absorption capacity in sunscreen cosmetics [1]. TiO_2_ is also used in the composition of dental prosthesis and implant biomaterials. Because of these multiple applications, TiO_2_ is massively produced and as nano-sized particles is found in variable proportions in daily life products. For example, for food grade TiO_2_ (E171) fraction of particles under 100 nm, thus corresponding to the strict definition of NPs was estimated at 36 % by Weir et al. and 5–10 % reported by Peters et al. [2, 3].

Recent toxicological and pharmacological research on rodents has shown that TiO_2_ NPs may translocate across physiological barriers such as respiratory, intestinal and vascular epithelium and therefore reach various organs and tissues including the brain [4–6]. The brain is well protected by the blood brain barrier (BBB), which contains cells of several types: brain endothelial cells (BECs), astrocytes, and pericytes [7–11]. These cells communicate closely to guarantee a physical and functional barrier between the blood and the central nervous system (CNS): tight junctions connecting endothelial cells and many transporters including two major ATP-driven drug efflux pumps, the P-glycoprotein (P-gp) and breast cancer resistant protein (BCRP) [12–14]. This neurovascular unit regulates distribution of xenobiotics to the brain.

Fabian et al. reported a lack of translocation of 20 nm TiO_2_ NPs to the brain parenchyma 24 h after intravenous (IV) injection of a dose of 5 mg/kg to rats [15]. These findings are further supported by other studies results in which lack of brain distribution was noticed 6 h following IV administration of 0.95 mg/kg of Degussa P25 TiO_2_ NPs to rats [16] and 24 h after administration of a 10 mg/kg dose of rutile TiO_2_ NPs to mice [17]. However, Geraets et al. showed the presence of TiO_2_ NPs of different sizes and crystalline forms 24 h after a 5 mg/kg IV dose in the brain of rats [18]. While brain translocation of TiO_2_ NPs after IV administration remains contradictory, oral and pulmonary exposure in rodents has shown distribution of TiO_2_ NPs to the CNS [18, 19]. Disturbance of neurotransmitters and enzymes, oxidative stress and inflammatory response have been described as neurotoxic effects after nasal instillation, intraperitoneal injection, oral administration or prenatal exposure [20–25]. These findings raised the question of the entry of TiO_2_ NPs into the brain and of their interactions with the BBB.

Our previous data using an *in vitro* BBB model [26] show that P25 TiO_2_ NPs (21.5 nm, 75 − 25 % anatase/rutile) can accumulate in BECs, with very low and limited translocation in the glial cells, hinting at the “physical barrier” role of the BEC epithelium [27]. Overall, these contradictory *in vitro* and *in vivo* findings show that additional investigations are needed to ascertain *in vivo* brain translocation of TiO_2_ NPs, interactions between TiO_2_ NPs and the BBB, and potential adverse effects in the brain. These are the main subjects of the present study.

We used well-characterized anatase/rutile TiO_2_ NPs (P25 aeroxide Degussa) to evaluate *in vivo* biodistribution of TiO_2_ NPs, with particular emphasis on *in vivo* interactions with the BBB. We evaluated the biokinetics of TiO_2_ NPs from 5 min up to one year after IV administration to adult Fischer rats at a dose of 1 mg/kg. This mode of administration and concentration was chosen for comparison to the contradictory literature. Moreover, IV allows clarify the potential brain translocation under conditions of 100 % systemic bioavailability without impact of NPs transport across other biological barrier that could impact NPs. In addition, the potential effects on BBB physiology and subsequent induction of neuroinflammation markers were analyzed.

## Results and discussions

### Characterization of titanium dioxide nanoparticles

**Fig. 1:**
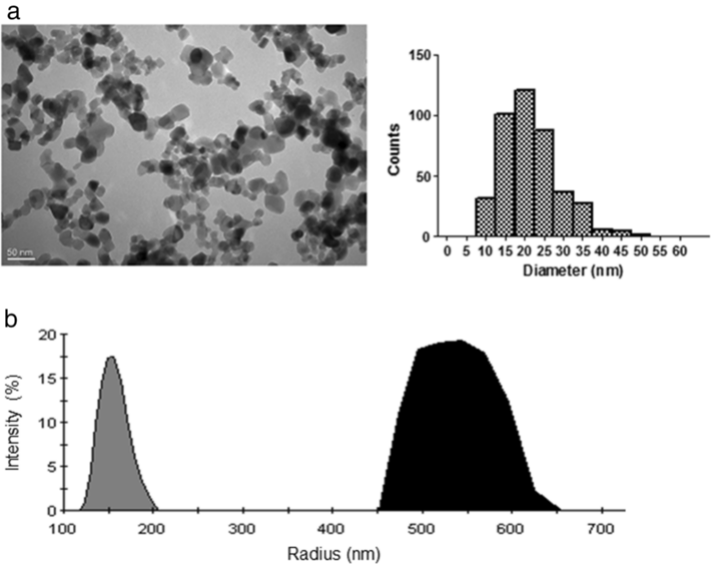
Characterization of TiO_2_ NPs. **a** Representative TEM image of the TiO_2_ NPs (magnification 40.000 x) and size distribution histogram. More than 400 NPs were measured randomly on several micrographs. The average diameter was 21.5 nm and the standard deviation 7.2. **b** Size distribution by intensity obtained by DLS for TiO_2_ NPs suspended in water (grey) or in saline buffer (black). Vigorous vortexing was the only treatment of the suspensions and measurement conditions were optimized resulting in a fixed lens position at 4.65 mm and a concentration of 25 μg/mL. Mean hydrodynamic radius were 163.5 ± 12.6 and 520.9 ± 41.7 nm for water and saline buffer respectively

Morphology and size of TiO_2_ NPs in stock suspensions were determined by Transmission Electron Microscopy (TEM). The mean diameter of individual particles was found to be 21.5 ± 7.2 nm (400 counts) (Fig. 1a). Scanning Electron Microscopy (SEM) confirmed spherical morphology of particles and Energy Dispersive X-ray (EDX) analysis on the same images confirmed purity of the product (i.e. only Ti and O are detected indicating the absence of other metal contamination greater than 1 %) (Data not shown). Analysis by X-ray diffraction confirmed the presence of a mixture of 75 % anatase and 25 % rutile crystal phases (Data not shown). The specific surface area, 51 m^2^/g, was measured by Brunauer–Emmett–Teller (BET) method and corresponds to 30 nm grain diameter.

Dynamic light scattering (DLS) measurements shows that NPs agglomerate in water and to a larger extent in saline buffer with hydrodynamic diameters of 163.5 ± 12.6 and 520.9 ± 41.7 nm, respectively (Fig. 1b). Those measurements highlight the important tendency of TiO_2_ NPs to aggregate/agglomerate as soon as they are in suspension. We choose not to apply other dispersion protocol for *in vivo* experiments as they usually necessitate modifying NPs surface. In addition, the size distribution we obtained is comparable to the one in Fabian, Xie and Geraets’s study [15, 17, 18] which would ease data confrontation. Indeed, it is worth noticing that even agglomerated in suspension, NPs form presents a greater reactive surface area that may promote adverse effect compare with the same material particles in micro size scale.

### Biodistribution and accumulation studies of titanium dioxide nanoparticles

**Fig. 2:**
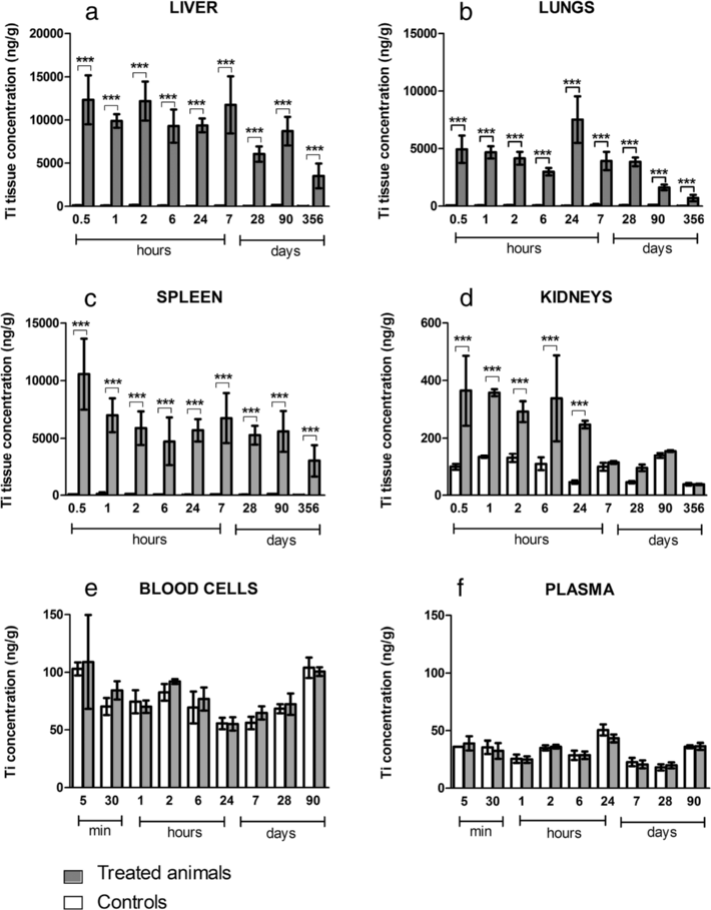
Tissue distribution of titanium after IV injection of 1 mg/kg TiO_2_ NPs in rats. Titanium quantification in liver (**a**); lungs (**b**); spleen (**c**); kidneys (**d**); blood cells (**e**) and plasma (**f**) of treated (*grey*) and control (*white*) animals. Quantification by inductively coupled plasma mass spectrometry (ICP-MS). Each data point represents the mean ± SD of n = 6 animals. Statistical comparison was performed by two-way ANOVA, *P < 0.05; **P < 0.01; ***P < 0.001

Ti burdens in organs and tissues after IV administration are shown in Fig. 2. In a previous work, we fully compared four commonly used mineralization methods for TiO_2_ NPs and established that one of them involving nitric and hydrofluoric acids, combined to the inductively coupled plasma mass spectrometry (ICP-MS) method allows Ti quantification for NPs in biological samples with limit of quantification (LOQ) from 4.7 to 33.1 ng/g depending on the tissue sample mass available. This method was validated for linearity, repeatability and accuracy. Matrix effects and recoveries were checked and quantification limit was determined, *sine qua none* conditions for providing valuable data (Devoy et al., 2015 accepted paper). Ti burdens in liver, spleen and lungs of the treated group were significantly higher than those of the control group from 30 min to 356 days (P < 0.001). The Ti level was higher in the liver than in spleen and lungs. Assays performed one year after TiO_2_ NPs administration to rats indicated that Ti burden remained high, suggesting long-term biopersistence and no major elimination from the liver. Indeed, approximately 33 % of the Ti burden at early time points (less than 24 h) remain in the liver one year after IV administration. The Ti burden in kidneys of the treated group was significantly higher from 30 min to 24 h, and then decreased significantly after 7 days after IV administration. Significant levels of Ti were never found in blood (plasma or blood cells) of the treated group, even at early end point (i.e. 5 min).

Our findings confirm previous studies showing that the liver, spleen and lungs appear to be the major organs of accumulation of TiO_2_ NPs after IV administration [15–18, 28] in rodents. We estimate that we recover approximately 44 % of the administered dose in the liver, 10 % in lungs and 2 % in spleen 6 h after IV administration. As reported previously, the clearance of TiO_2_ NPs from the blood circulation is rapid, so Ti was never detected in plasma or blood cells [15, 28]. The limit of quantification of the ICP-MS method used is about 15 ng/ml for plasma and about 14 ng/g for blood cells. In this context a low blood concentration of Ti cannot be ruled out. Driven by the quantification method developed, we then focused on the Ti brain content.

### Quantification of titanium dioxide in the brain

**Fig. 3:**
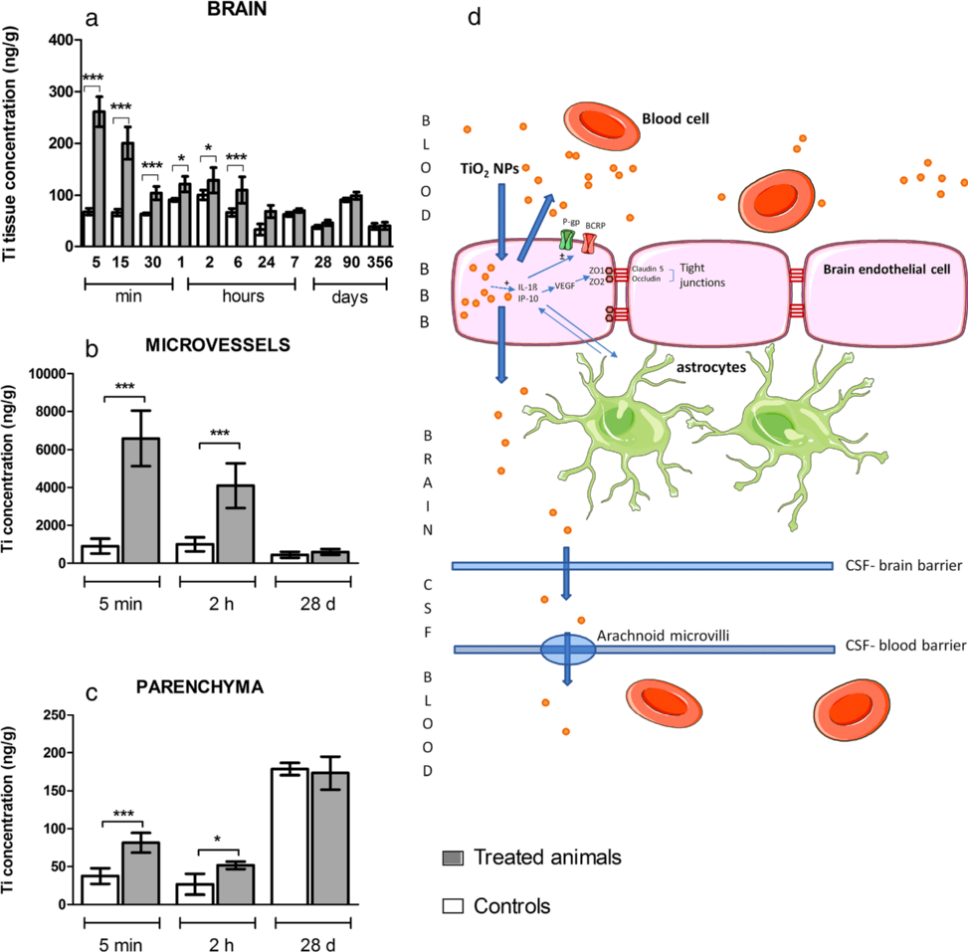
Titanium quantification in rat brain (**a**), isolated brain microvasculature endothelial cells (**b**) and brain parenchyma (**c**) of treated (*grey*) and control (*white*) animals after IV injection of 1 mg/kg TiO_2_ NPs and schematic summary of the events and potential effects on the BBB.. Quantification by inductively coupled plasma mass spectrometry (ICP-MS). Each data point represents the mean ± SD of n = 6 animals. Statistical comparison was performed by two-way ANOVA, *P < 0.05; **P < 0.01; ***P < 0.001. Consequences and hypothesis of clearance mechanism of TiO_2_ NPs at the BBB (**d**). Transient presence of TiO_2_ NPs in brain microvasculature endothelial cells leads to overexpression of cytokines and chemokines (IL-1β and IP-10), which could be linked to the modification of activity or expression of P-gp and BCRP. Integrity of the barrier is not compromised

IV administration of TiO_2_ NPs to rats resulted in trace amount of Ti content in total brain at early end points (Fig. 3). Ti was significantly detected from 5 min (brain Ti concentration was 261.40 ± 28.86 ng/g for the treated group vs. 68.25 ± 6.56 ng/g for the control group, *P* < 0.001) to 6 h (brain Ti concentration was 110.00 ± 25.05 ng/g for the treated group vs. 66.75 ± 7.36 ng/g for the control group, P < 0.001) (Fig. 3a). We found a significant decrease in the cerebral tissue Ti concentration after 24 h. Ti content after 24 h in the treated group did not differ significantly from that in the control group. These data are in agreement with the recent study by Geraets et al., who found Ti in rat total brain 24 h after IV administration of 5 mg/kg doses of different TiO_2_ NPs whatever their sizes and crystallinity [18]. Shinohara *et al.* also measured traces of Ti in rat total brain 6 h after IV administration of a 0.95 mg/kg dose of P25 Degussa TiO_2_ NPs [16]. Fabian et al. did not detect Ti in the total brain, a result which could be explained by the sensitivity of the analytical inductively coupled plasma atomic emission spectroscopy (ICP-AES) method used.

These observations raise one important question: do TiO_2_ NPs cross the BBB and accumulate in the brain parenchyma or remained located in the brain micro vasculature? As a crucial component of the BBB, the BECs and a fraction corresponding to glial and neural cells referred to as the parenchyma fraction were separated, as we described previously [29]. The purity of the resulting BECs was checked after RNA isolation and real-time PCR experiments by measuring the expression of cell-specific marker genes for BECs (CD31 or PECAM) and for glial cells (glial fibrillary acid protein). The purity of isolated BECs was 99.58 ± 0.11 % and of the parenchyma fraction 98.52 ± 1.17 %. Analysis of Ti content in these two separated fractions highlighted rapid internalization of TiO_2_ NPs into BECs (at 5 min after IV Ti concentration in treated group was 6579.33 ± 594.36 ng/g vs 907.16 ± 158.70 ng/g in the control group *P* < 0.001), followed by a significant decrease at 2 h (Fig. 3b). With caution to the background increased in the untreated animals at 28 days, at 5 min and 2 h after IV a significant increase of Ti between controls and treated animals was found in the parenchyma fraction but correspond to a very low amount of Ti (Fig. 3c). At 28 days after IV administration, Ti content in BECs was not different in the control and treated groups as well as in the parenchyma fraction of both same age groups. Notwithstanding the uptake of TiO_2_ NPs by the BECs and the very low concentration found in brain parenchyma, our findings suggest that the BBB regulates the uptake and clearance of TiO_2_ NPs. This is in line with previous *in vitro* findings from our laboratory that have demonstrated rapid internalization of TiO_2_ NPs by BECs [27] wherein NPs have been evidenced in vesicles, suggesting that isolated or aggregated NPs can be exported from the cell *via* exocytosis. *In vivo*, two clearance pathways can be hypothesized: exocytosis back in the blood circulation or transcytosis across BECs to enter the cerebrospinal fluid (Fig. 3d). The mechanism of internalization and clearance of Ti from the BECs thus remain to be determined.

### BBB physiology modulations

Here we have shown that there is early clearance of TiO_2_ NPs at the BBB and significant biopersistence of Ti in organs (i.e. liver, spleen, etc.) after IV administration of TiO_2_ NPs to rats. This raises the question of whether there is a direct or indirect effect of TiO_2_ NPs on BBB physiology in terms of BBB integrity, on regulation of proteins involved in brain detoxification, such as P-gp and BCRP, and regulation of neuroinflammation.

#### BBB integrity

Since alterations in the expression and/or distribution of tight junction proteins are associated with pathophysiological conditions, such as neurological disorders (Alzheimer disease, multiple sclerosis, dementia, epilepsy, etc.) [10, 30–32], we investigated whether the presence or lack of TiO_2_ NPs in the BECs after IV administration to rats would compromises BBB integrity or not. Occludin and claudins are key proteins in tight junctions that seal neighboring BECs and limit paracellular diffusion of substances. The expression of claudin-5 and occludin mRNA was determined, as well as the partition coefficient or Kp of atenolol, a known paracellular drug marker [33], which does not cross the BBB in normal physiological condition. The marketed increase of atenolol Kp will reflect the increased in the apparent permeability of atenolol attributed to the BBB disruption.

**Fig. 4:**
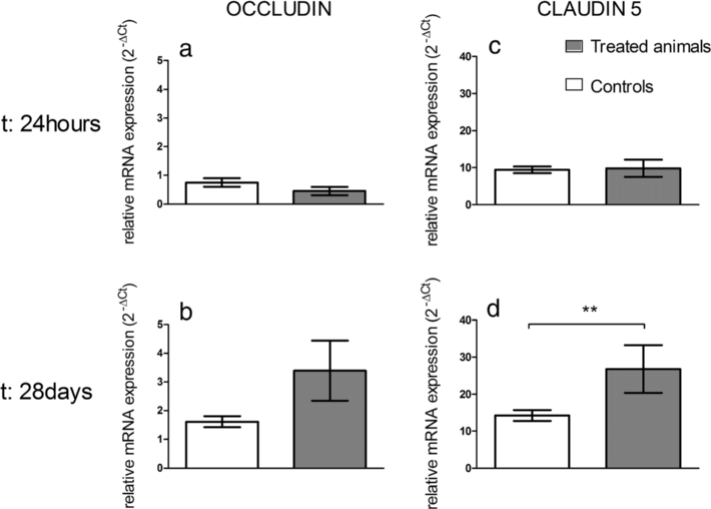
mRNA expression of tight junction proteins occludin (**a, b**) and claudin 5 (**c, d**) in isolated brain microvasculature endothelial cells 24 h or 28 days after IV injection of 1 mg/kg TiO_2_ NPs in rats. RT-qPCR was performed in duplicate for each single brain microvasculature endothelial cells preparation. Each data point represents the mean ± SEM of n = 4 animals. Statistical comparison was performed by two tailed Mann-Whitney test, ** *P* < 0.01. For occludin mRNA expression *P* = 0.057

**Fig. 5:**
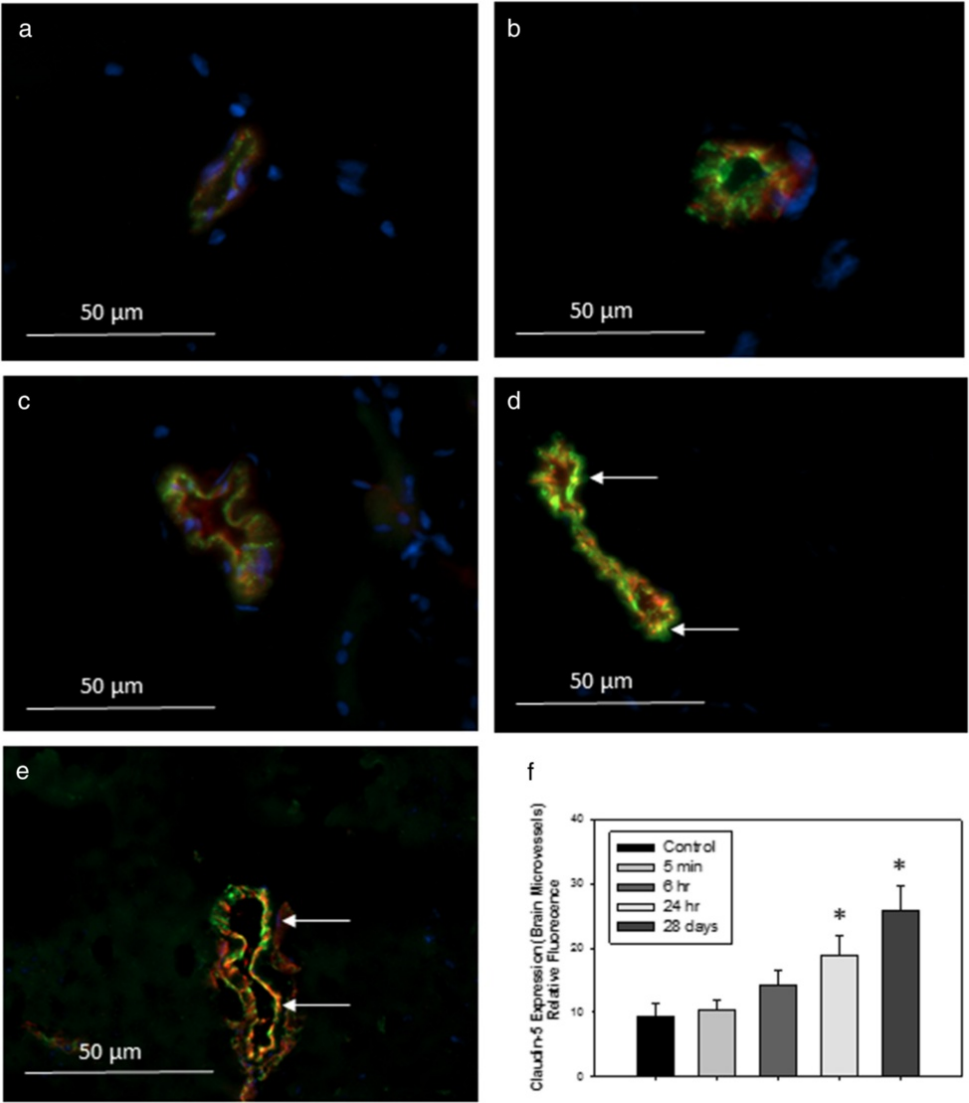
Representative images of immunofluorescence expression of tight junction protein claudin-5 (*red*) and endothelial cell marker vonWillebrand factor (vWF, *green*) in the cerebral microvasculature of (**a**) control rats, or tissues collected from rats treated with 1 mg/kg TiO_2_ (IV) at (**b**) 5 min; (**c**) 6 h; (**d**) 24 h or (**e**) 28 days post-treatment. Yellow fluorescence indicates overlay (co-localization) of claudin-5 and vWF. Arrows indicate areas of increased yellow fluorescence. Graph in (**f**) shows analysis of relative fluorescence of overlay images ± SD. *P < 0.050 compared to controls

**Fig. 6:**
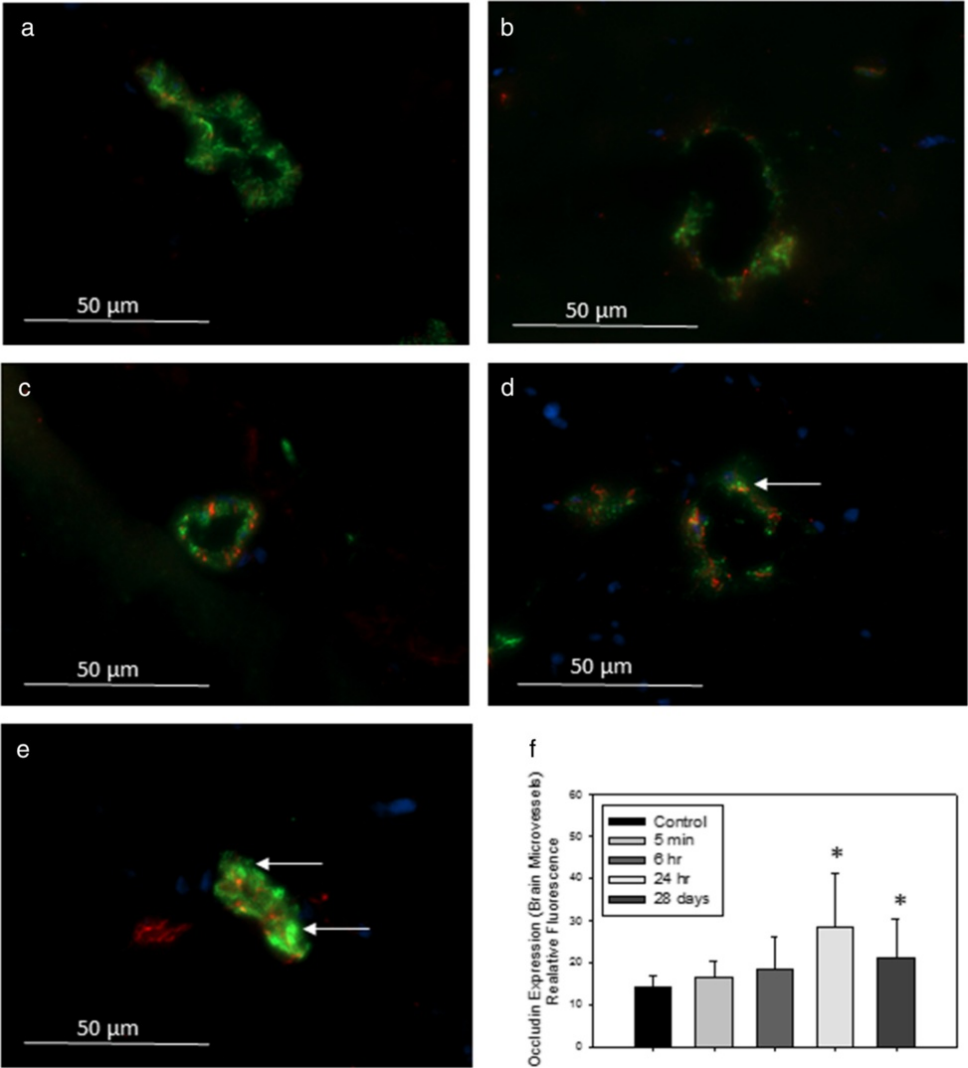
2Representative images of immunofluorescence expression of tight junction protein occludin (*red*) and endothelial cell marker vonWillenbrand factor (vWF, green) in the cerebral microvasculature of (**a**) control rats, or tissues collected from rats treated with 1 mg/kg TiO_2_ (IV) at (**b**) 5 min; (**c**) 6 h; (**d**) 24 h; or (**e**) 28 days post-treatment. Yellow fluorescence indicates overlay (co-localization) of occludin and vWF. Arrows indicate areas of increased yellow fluorescence. Graph in (**f**) shows analysis of relative fluorescence of overlay images ± SD. * *P* < 0.050 compared to controls

**Fig. 7:**
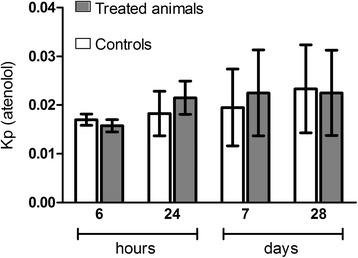
BBB integrity assessment. The integrity was estimated by the ratio between atenolol concentrations in brain and plasma (partition coefficient or Kp) at 6 h, 24 h, 7 days and 28 days after IV administration. Each data point represents the mean ± SD of n = 4 rats. Statistical comparison was performed by two-way ANOVA

The expression profiles of the genes encoding claudin-5 and occludin are represented in Fig. 4. In freshly isolated BECs, their mRNA expression levels were not significantly different between control and treated animals at 24 h after IV administration of TiO_2_ NPs (Fig. 4a and c). However, notwithstanding the lack of Ti within the brain, the biopersistence of Ti in the other organs revealed an increase of claudin-5 and a slight increase for occludin mRNA expression (*P* = 0.057) in the BECs 28 days after IV administration of TiO_2_ NPs (Fig. 4b and d). The upregulation of tight junction mRNA in BECs correlates with protein expression evidenced by immunofluorescence staining (Figs. 5 and 6). In addition, the brain to plasma concentration ratio (Kp) of atenolol was determined. The atenolol Kp between controls and treated animals remained unchanged (Fig. 7), suggesting lack of BBB breakdown after administration of 1 mg/kg TiO_2_ NPs in rats. However, higher doses of TiO_2_ NPs (10 mg/kg) induced an increase of atenolol Kp 6 h after IV administration, suggesting compromise of BBB integrity and testifying for the ability of the integrity probe (Additional file 1). These findings on the consequences of exposing the BBB to TiO_2_ NPs are in accordance with observations on the *in vitro* BBB model (Additional file 2).

Overall, these data suggest BECs activation at 28 days and a plausible establishment of a BBB repair mechanism after IV administration of TiO_2_ NPs (1 mg/kg). This raises the question of the mediators potentially involved in these processes.

#### Regulation of P-gp and BCRP at the BBB

**Fig. 8:**
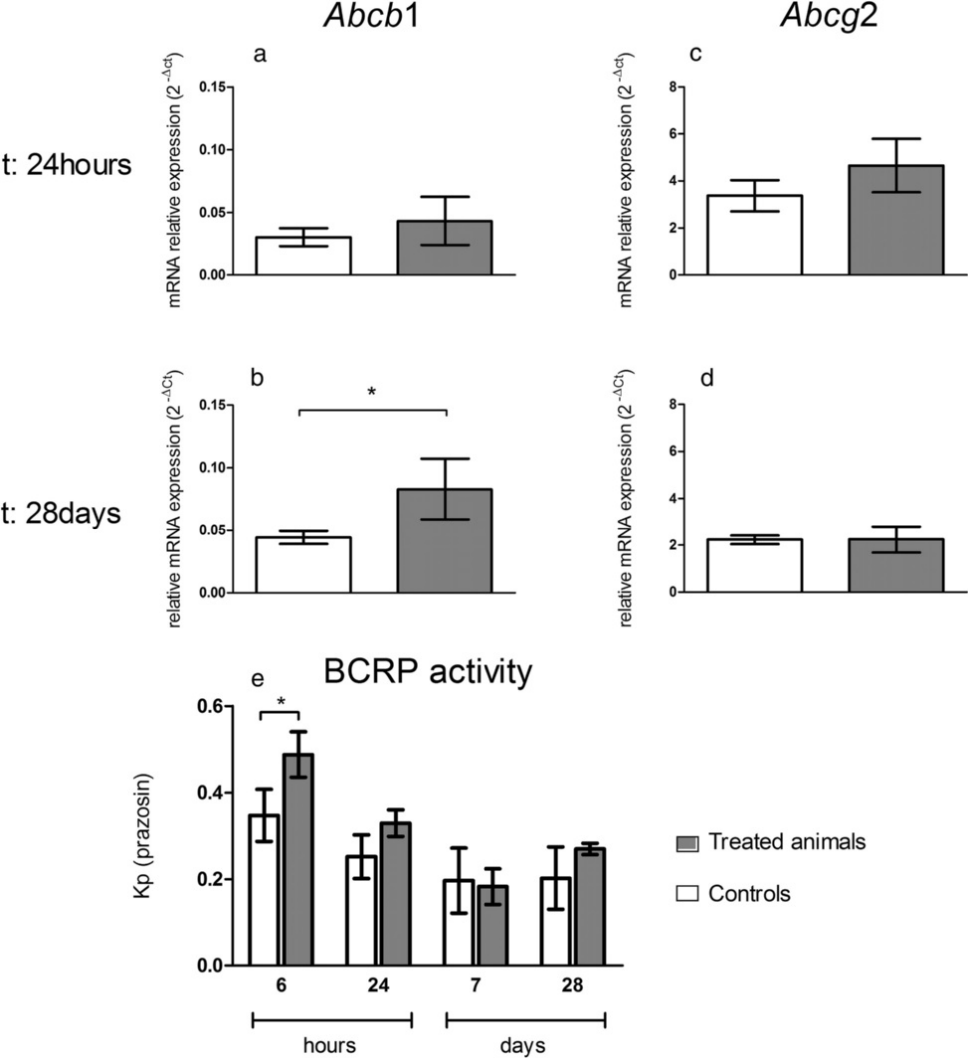
mRNA expression of transporters *Abcb1* (P-gp) and *Abcg2* (BCRP) transporters in isolated brain microvasculature endothelial cells and BCRP activity assessment at the BBB after IV injection of 1 mg/kg TiO_2_ NPs in rats. mRNA transcription profiles for *Abcb1* (**a; b**) and *Abcg2* (**c; d**) were performed by RT-qPCR in isolated rat brain microvasculature endothelial cells of control and treated animals. Each data point represents the mean ± SEM of n = 4 animals and RT-qPCR was performed in duplicate for each single preparation of brain microvasculature endothelial cells. Statistical comparison was performed by two tailed Mann-Whitney test, *P < 0.05. BCRP activity (**e**) was estimated by the ratio between prazosin concentrations in brain and plasma (partition coefficient or Kp). Each data point represents the mean ± SD of n = 4 rats. Statistical comparison was performed by two-way ANOVA, *P < 0.05

A number of transport and carrier systems are expressed and polarized on the luminal or abluminal surface of the BECs. Among these systems, Adenosine triphosphate-Binding Cassette (ABC) transporters play a critical role in preventing neurotoxic substances from entering the brain, and in transporting toxic metabolites out of the brain. mRNA expressions as well as transport activities of two major ABC transporters (*Abcb1*/P-gp and *Abcg2*/BCRP) at the BBB were investigated after IV administration of TiO_2_ NPs. Data depicted in Fig. 8a and c show no change in *Abcb*1 and *Abcg2* mRNAs at 24 h in the rat BECs, while biopersistence of Ti in organs except brain (28 days) correlated with an increase in *Abcb1* mRNAs but not *Abcg2* mRNAs expression (Fig. 8b, d), suggesting differential P-gp and BCRP transporter regulation mechanisms. The regulation of *Abcb1* mRNAs has been also described in the context of oxidative stress, signaling initiated by Diesel Exhaust Particles for example, due to the activation of Nicotinamide Adenine Dinucleotide Phosphate-oxidase which produces Reactive Oxygen Species, stimulates Tumor Necrosis Factor - α (TNF-α) release and activate TNF receptor 1 (TNF-R1). In turn, TNF-R1 activates the transcription factor Activator Protein 1 leading to an increase in P-gp expression. Activation of nuclear factor E2-related factor-2, a sensor of oxidative stress, also upregulates P-gp expression at the BBB [34]. To determine whether the increase in mRNA expressions resulted in changes in protein activity, we measured the Kp of digoxin and prazosin as examples of P-gp and BCRP substrates [35–39], respectively. A decrease of transporter activity is indicated by his substrate Kp increase. Functional studies show a rapid down regulation of BCRP activity indicated by a brain increase of prazosin concentration 6 h after exposure to TiO_2_ NPs (Kp_prazosin_ = 0.48 ± 0.03 for treated group versus Kp_prazosin_ = 0.34 ± 0.03 for control group), whereas no change in transport activity was observed at 24 h, 7 or 28 days after TiO_2_ NP exposure (Fig. 8e). These findings pointed out different signaling processes at the BBB level during early (e.g. direct interaction of TiO_2_ NPs with the BBB) and late events (e.g. cytokine signaling) after IV TiO_2_ NPs administration to animals. Such regulation has been evidenced when brain capillaries was exposed to low levels of Lipopolysaccharide [34, 40]. We could not correlate the increase in *Abcb1* mRNA expressions 28 days with the P-gp protein transport activity since brain digoxin concentrations were under the quantification limit in both the controls and the treated group, so P-gp activity was not measurable. It is worth noticing that this disturbance of P-gp and BCRP expression or activity was also observed on the *in vitro* cell based BBB model after 24 h exposure to TiO_2_ NPs (data not shown). Such disturbance of BCRP or P-gp activities could alter the detoxification function of the BBB. Indeed, P-gp and BCRP have a very wide variety of substrates and modulation of their efflux capacities even low can potentially lead to accumulation of neurotoxics in the brain parenchyma [41].

### Neuroinflammation assessment and relation to biopersistence of TiO_2_ NPs in organs

**Fig. 9:**
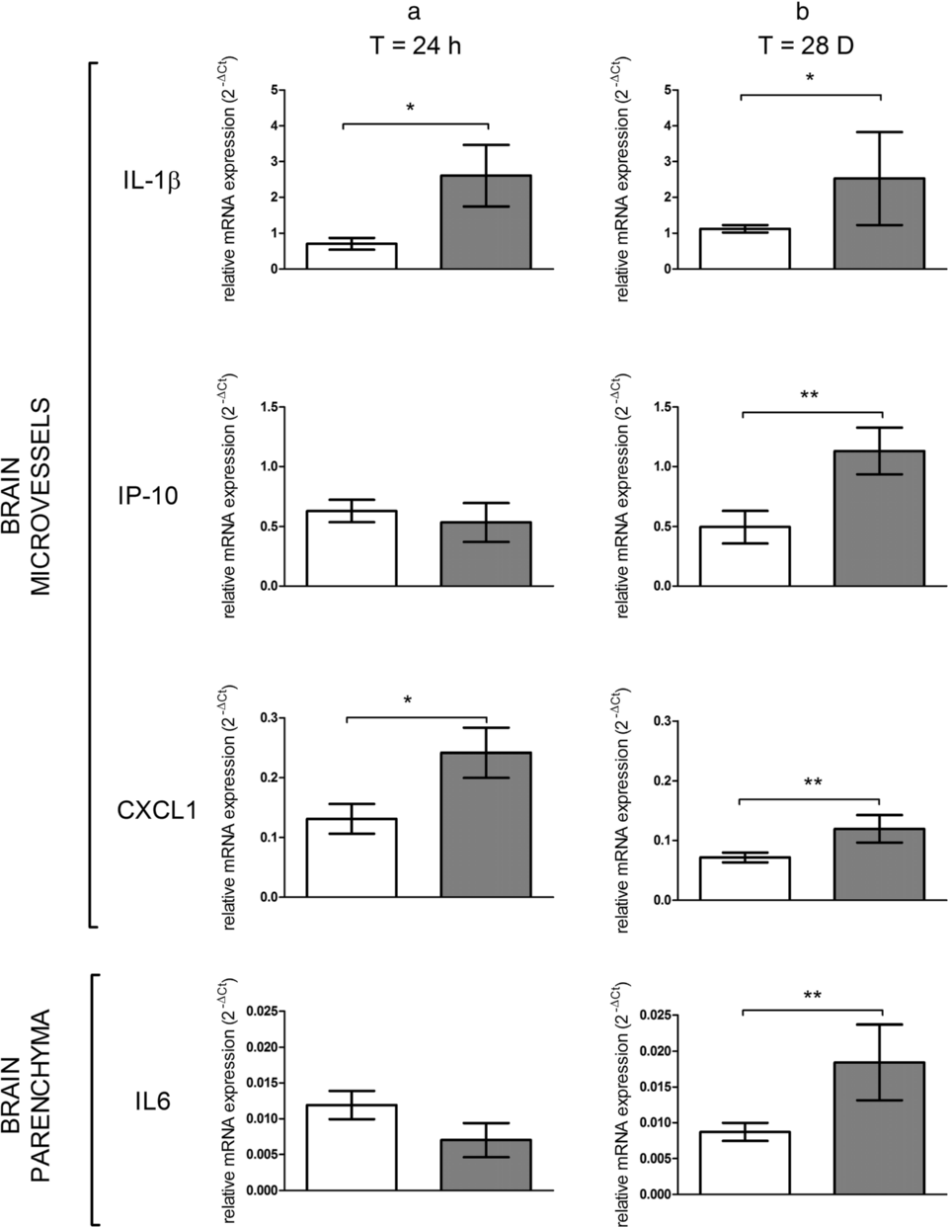
mRNA expression of cytokines and chemokines in isolated brain microvasculature endothelial cells and parenchyma fraction after IV injection of 1 mg/kg TiO_2_ NPs in rats. mRNA transcription profiles for IL-1β, IP-10 and CXCL1 in isolated rat brain microvasculature endothelial cells and IL6 in parenchyma fractions of control and treated animals performed by RT-qPCR at 24 h (**a**) and 28 days (**b**) after IV injection. Each data point represents the mean ± SEM of n = 4 animals and RT-qPCR were performed in duplicate for each single preparation of brain microvasculature endothelial cells. Statistical comparison was performed by one tailed Mann Whitney test, *P < 0.05; ** *P* < 0.01

**Fig. 10:**
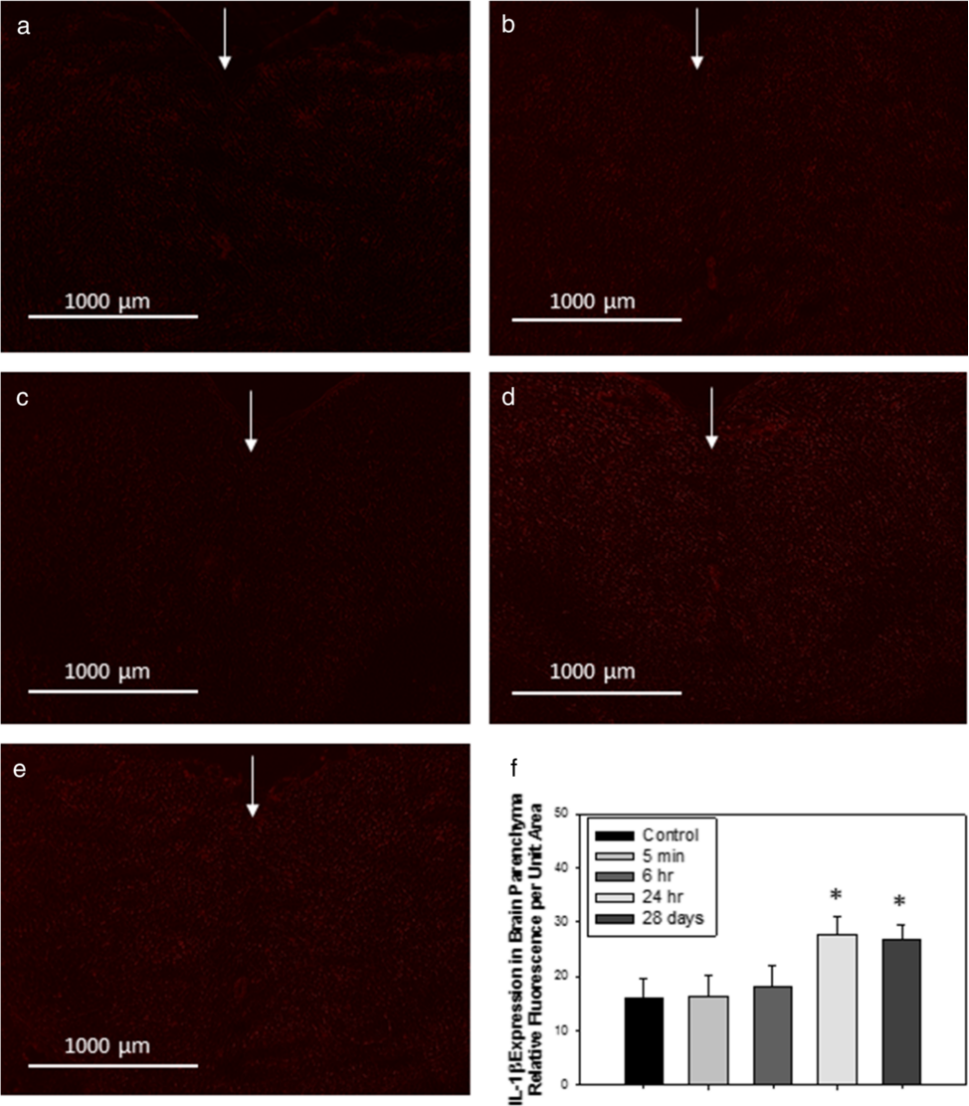
Representative images of immunofluorescence expression of IL-1β in the cerebral parenchyma of (**a**) control rats, or tissues collected from rats treated with 1 mg/kg TiO_2_ (IV) at (**b**) 5 min; (**c**) 6 h; (**d**) 24 h; or (**e**) 28 days post-treatment. Arrows indicate sagittal suture (coronal cut, sections cut and analyzed between rostral and caudal diencephalon). Graph in (**f**) shows analysis of relative fluorescence of overlay images ± SD. *P < 0.050 compared to controls

Finally, we were concerned about a potential neuroinflammation as such a phenomenon was at stage in our *in vitro* cell based BBB model and described *in vivo* after intraperitoneal injection of TiO_2_ NPs [22, 42]. We studied the influence of the short-term presence of Ti in the BECs and the impact of biopersistence of Ti in other organs on CNS inflammation, especially at the BBB and in the brain parenchyma. The presence of Ti in BECs at early end point depicted in Fig. 3b (significant up to 24 h after IV administration) is correlated with a significant increases of interleukin 1β (IL-1β) and chemokine ligand 1 (CXCL1) (Fig. 9). These increases in addition to γ inducible protein-10 (IP-10/CXCL10) are maintained 28 days after IV administration, whereas no Ti was detected in BECs or in the brain parenchyma. In the brain parenchyma, we also noted an increase of interleukin 6 (IL6) expressions at 28 days after IV administration (Fig. 9). First, the persistent brain inflammation evidenced in this study at the brain microvasculature level 28 days after TiO_2_ NPs exposure raised the question of potential brain dysfunction. Indeed, a link between persistent neuro-inflammation and brain pathologies as already been established [43]. Second, modifications of P-gp and structural tight junction protein mRNA expressions come with modulation of cytokines and chemokines 28 days after TiO_2_ NPs administration to rats. This is not correlated with measurable Ti brain and plasma content. In this context, an indirect mechanism of interactions between NPs and BECs could be suggested. It is likely that soluble mediators interacts with their BBB targets and elicit BECs activation and neurovascular inflammation. For example, pro-inflammatory mediators such as TNFα distributed through the systemic circulation affect ABC transporter expression and activity [44, 45]. We thus suggest that 28 days after exposure, circulating cytokines and chemokines released probably by organs containing high amounts of Ti induce activation of BECs and initiate the release of IL-1β, IP-10 and CXCL1, which act in a paracrine way to activate astrocytes/microglia cells in the brain parenchyma. Immunofluorescence staining revealed an increase of IL-1β in the rostral and caudal diencephalon (Fig. 10). Because of its massive and biopersistence of Ti, liver could be a key organ for induction of oxidative stress, lipid composition modifications, and immune response as reported elsewhere [25, 46–48]. Mediators such as cytokines, chemokines and lipids circulating in the blood may be responsible indirectly for BBB deregulation at late end points. Transposition of the effect evidenced at the BBB on other endothelial cells by blood circulating factors could not be excluded.

### Circulating serum factors from rats treated with TiO_2_ NPs induce an inflammatory response in a cell-based BBB model

To demonstrate whether circulating cytokines/mediators that may be released by organs bioaccumulating Ti can lead to neurovascular inflammation and BBB physiology alterations, we performed *in vitro* studies on the primary rat cell-based BBB model described previously [26]. The model was exposed to diluted sera from untreated and treated animals collected 28 days after exposure to TiO_2_ NPs. Exposure of the apical compartment mimics blood-borne exposure. After 24 h of exposure to serum, the integrity of the BECs monolayer was checked and BECs and glial cells were recovered for mRNA profiling.

**Fig. 11:**
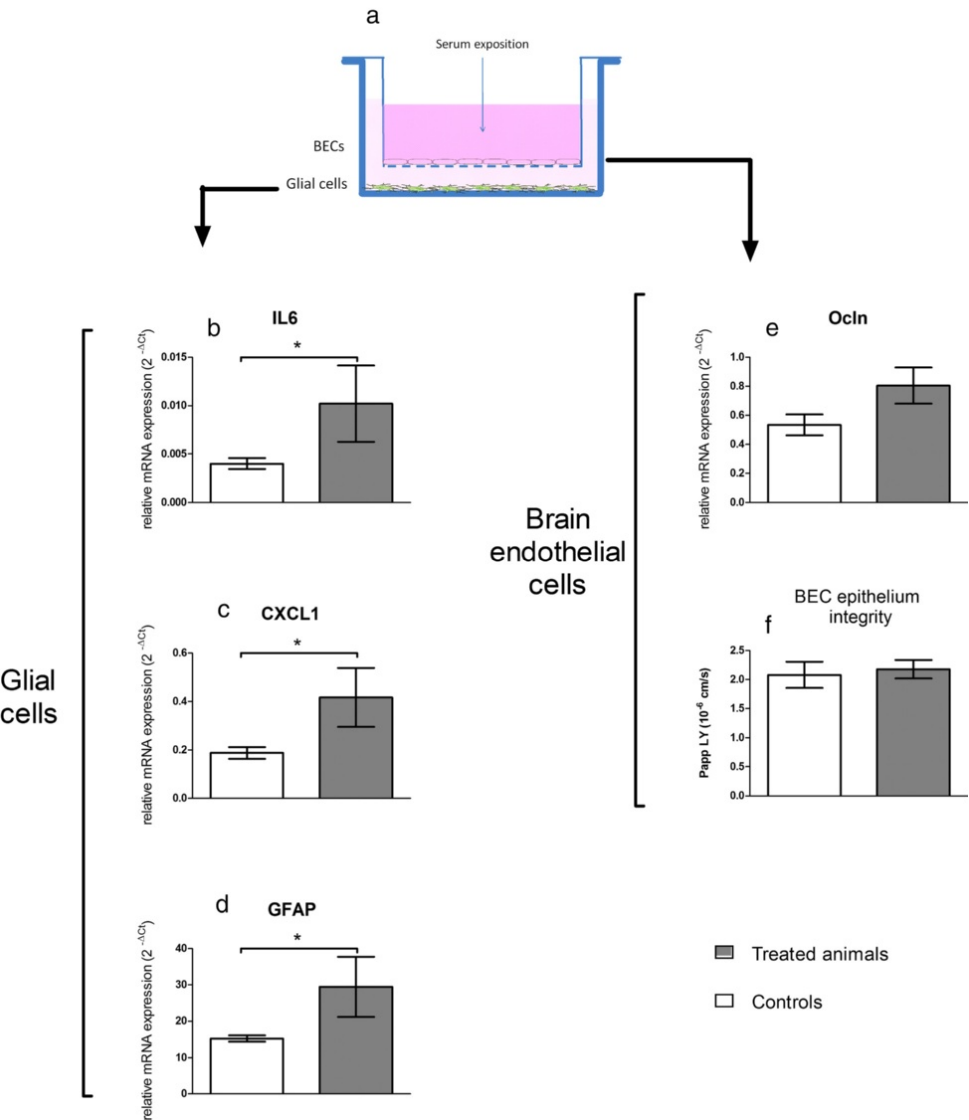
Impact of exposure to sera from treated animals on the *in vitro* BBB model. (**a**) is a schematic view of the model architecture. BECs were grown on a semipermeable membrane whereas glial cells lay on the well bottom. mRNA expressions were quantified by RT-qPCR after 24 h exposure to sera from control and treated rats. For glial cells, expressionsof cytokines and chemokines (IL6 (**b**), CXCL1 (**c**)) and of GFAP (**d**) are presented. For BECs, tight junction protein occludin (Ocln (**e**)) is reported and integrity of the BECs monolayer was checked in terms of apparent permeability to Lucifer yellow (**f**). Sera diluted in culture medium were applied to the BECs compartment for 24 h. Each data point represents the mean ± SEM of n = 4 samples, each sample representing the mRNA pool from 6 wells. RT-qPCR was performed in duplicate for each single sample. Statistical comparison was performed by one tailed Mann Whitney test, * *P* < 0.05 for IL-6, CXCL1 and GFAP mRNA expressions. Statistical comparison was performed by two tailed Mann Whitney test for Ocln mRNA expression (*P* = 0,057)

After 24 h of exposure, the integrity of the BECs monolayer remained intact in the control group as well as in the treated group (Fig. 11f), thus confirming the maintenance of BBB integrity observed 28 days after exposure *in vivo* (apparent permeability (P_app_) for Lucifer Yellow (LY) was 2.08 ± 0.99 cm/s for controls vs 2.17 ± 0.77 cm/s for the treated group). In BECs, we noted a tendency of increase for occludin mRNA expression (*P* = 0.057), which is in accordance with *in vivo* observations. The mRNA expressions of IL6, CXCL1 and glial fibrillary acid protein (GFAP) in astrocytes were upregulated. These inflammatory markers are key proteins for communication between glial and BECs [49–54]. This *in vitro* mRNA upregulation in glial cells of the BBB model suggests that the mediators are present 28 days after exposure in the serum of treated rats and allows to consolidate our hypothesis of circulating mediators.

## Conclusions

**Fig. 12:**
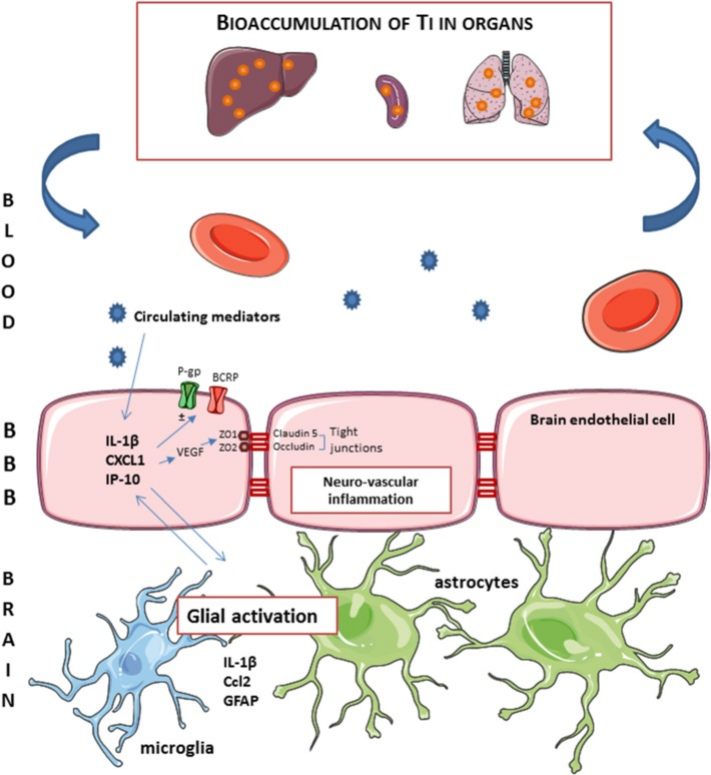
Schematic summary of the events and potential effects on BBB physiology in the case of bioaccumulation of TiO_2_ NPs in organs. Exposure to circulating mediators that maybe originate from organs bioaccumulating titanium leads to neurovascular inflammation. Overexpression of IL-1β, CXCL1 and IP-10 may contribute to modification of brain endothelial cell physiology in terms of expression of tight junction proteins (occludin and claudin-5) and ABC transporters (P-gp and BCRP). In parallel, key inflammatory markers for glial and endothelial cells interactions were upregulated

The major findings of our study are depicted in Fig. 12 and Fig. 3d. They show a Ti burden in the liver, spleen and lungs up to 356 days after IV administration of TiO_2_ NPs to rats, with very low clearance rate observed until one year after administration. Additionally, we describe for the first time the *in vivo* uptake and clearance by BECs of TiO_2_ NPs after exposure. Furthermore, this is the first study reporting the link between deregulation of BBB physiology and the presence of TiO_2_ NPs in distal organs. Upregulation of tight junction proteins, modulation of P-gp mRNA expression and persistent brain inflammation markers such as IL-1β, IP-10 and CXCL1 were highlighted. Thus, regardless of where the peripheral signal originates from, our findings raise the question of circulating biomarkers potentially released by organs accumulating Ti to promote dysregulation of BBB physiology and neuroinflammation. Substantial research remains to be done to identify such peripheral biomarkers. Our findings point out for the first time that TiO_2_ NPs can exert indirect effect on the CNS that seems dependent on the circulation.

## Materials and methods

### Chemicals

Human serum, bovine serum albumin (BSA), N-alpha-tosyl-L-lysinyl-chloromethylketone (TLCK) and Lucifer yellow (LY), Digoxin, Prazosin, Atenolol and Atenolol-d7 were from Sigma Aldrich (Saint-Quentin Fallavier, France). Collagenase/dispase and DNAse I were from Roche Applied Science (Basel, Switzerland). Digoxien-d3 was from Artmolecule (Poitier, France).

All chemicals used for ICP-MS were of analytical grade. Nitric acid was used to prepare 0.2 % HNO_3_ (*v/v*) with ultrapure water. All single element stock solutions (1000 mg/L) were delivered by SCP Science and certified for purity and concentration. From these stock solutions, a mixed working standard solution with a concentration of 10 mg/L for each element was prepared by putting 1 mL of each stock solution in a 100 mL measuring flask, adding 5 mL of purified HNO_3_ and diluting to 100 mL with ultrapure water (MilliQ, Millipore, Germany).

### TiO_2_ nanoparticles

TiO_2_ P25 NPs (Aeroxide® P25, 75 % anatase 25 % rutile, Evonik®) were from Sigma Aldrich (Saint-Quentin Fallavier, France). For *in vivo* experiments, TiO_2_ NPs were suspended in sterile physiological salt solution at a stock concentration of 1 mg/mL. No other treatment was performed. A thorough characterization of NPs was conducted through a panel of complementary techniques. NPs were imaged by Transmission Electron Microscopy (TEM) with a JEOL 1400 instrument (JEOL, Tokyo, Japan) operating at 80 keV (Imagif platform, Gif-sur-Yvette) and Scanning Electron Microscopy (SEM) (Carl Zeiss, Ultra 55) equipped with an Energy Dispersive X-ray spectrometer (EDX) allowing elemental analysis. For both TEM and SEM analyses, samples were prepared as follows: 3 μL droplet of the dispersion was cast on formvar/ carbon-coated copper grids for 5 min. The Brunauer-Emmett-Teller (BET) method (Micromeritics FlowsorbII 2300) was applied to determine the specific surface area (SSA) of the nanopowder and diameter was calculated from the SSA value, as D = 6 000/(ρ.SSA), where D is BET diameter (nm), and ρ = 3.9 g.cm^−3^ is the density of anatase TiO_2_. The crystalline phases were measured by X-Ray Diffraction (XRD) with a Siemens D5000 instrument using the Cu-Kα radiation and using the Match software (crystal impact). Hydrodynamic diameter was measured by Dynamic Light Scattering (DLS) using a ZetaSizer ZEN3600 (Malvern, Herenberg, Germany) equipped with a 633 nm laser.

### Animals and intravenous TiO_2_ NPs administration protocol

Male Fisher F344 rats (from Charles River Laboratories, France), 8 weeks old and weight 180–250 g, were housed in standard environmental conditions (room humidity and temperature controlled 19 °C–23 °C; room under a 12 : 12 h light dark cycle) and maintained with free access to water and standard laboratory diet. The control animals were injected intravenously via the tail vein with 1 mL/kg of sterile saline buffer and the treated animals were injected with TiO_2_ NPs suspension at the dose of 1 mg/kg. TiO_2_ NPs suspension was vortex for 5 min before administration. No other dispersion protocol was used to avoid false positive results that would be due to inappropriate handling of the dispersion protocol of NPs.

30 min, 1 h, 2 h, 6 h, 24 h, 7 days, 28 days, 90 days or 356 days after IV injection, animals were anesthetized with isoflurane and euthanized. Additional time points at 5 min and 15 min was set up for brain and blood collection. Blood, liver, brain, spleen, kidneys and lungs were collected and stored at −80 °C until assayed. All procedures were approved by the CEA Institute’s Animal Care and Use Committee and conform to the Guide for the Care and Use of Laboratory Animals published by the European community’s council (directives 86/609/EEC, November 24, 1986), and the French directives concerning the use of laboratory animals (February 2013).

### Sample Preparation and Ti analysis by ICP-MS

Tissues from 6 controls and 6 treated rats were used for biodistribution studies. Tissues were thawed and about 0.1–0.3 g of each tissue was weighed, digested and analyzed for Ti content. Each sample was added to a 55 mL microwave digestion vessel along with 8 mL of nitric acid and 2 mL of hydrofluoric acid and digested using a Microwave Assisted Reaction System (MARS) Express instrument. The microwave digestion program was 15 min; 150 °C; 1200 W then 15 min; 180 °C; 1200 W followed by 20 min cooling. After cooling, the sample was rinsed 3 times using approximately 20 mL of 2 % nitric acid solution in a polytetrafluoroethylene (PTFE or Teflon) beaker. 2 mL of hydrogen peroxide was added to each beaker to digest any remaining organic matter. The beaker was then heated on a hot plate at 180 °C until between 0.1 and 0.5 mL of solution remained. The beakers were removed from the hot plate, allowed to cool and rinsed 3 times with 2 % nitric acid solution into a 25 mL volumetric flask before being stored for analysis. Samples were refrigerated at −20 °C while not in use.

This digestion method was evaluated for the recovery of a known amount of TiO_2_ and the ability to digest organic material sufficiently for analysis. The method was applied to a number of blank samples, containing only the reagents and no sample, in order to measure the amount of Ti contamination.

### Analysis of Ti with ICP-MS

Ti standard solutions for ICP-MS calibration were prepared at concentrations of 2, 4, 8, 10, 30, 50 and 100 ng/L, by diluting a 1 g/L Ti standard stock solution (1.70363.0100, SCP Science) with 2 % *v/v* HNO_3_ and 0.01 % *v/v* Triton X-100. An internal standard solution, containing 25 μg/L of Ge was prepared by diluting a 1000 mg/L internal standard stock solution (1.70320.0100, Merck) with 2 % *v/v* HNO_3_. The internal standard (25 μg/L Ge) was added in all samples and standards. Ti analysis of acidified samples was carried out using a Varian 820-MS. Samples in 2 % *v/v* HNO_3_ were directly analyzed with the Varian-820-MS for Ti determination.

### BBB permeability and P-Glycoprotein and BCRP transport activity measurement

Four hours before sacrifice, 4 control and 4 treated rats were subcutaneously implanted with mini-osmotic pumps (Alzet model 2001D; DURECT Corp., Cupertino, California). Pumps were filled with atenolol, digoxin and prazosin dissolved in PEG200/DMSO (50/50) to deliver at 0.25 mg/kg/h, 0.5 mg/kg/h or 0.25 mg/kg/h rates, respectively. The relevance of distribution study of atenolol, digoxin and prazosin in plasma and brain for BBB integrity and transport function assessment was previously demonstrated [26, 29]. 6 h, 24 h, 7 d or 28 d after IV injection, animals were anesthetized with isoflurane and euthanized. Plasma samples and brain were collected and weighed immediately after death. The administered substrates were quantified in the two compartments using mass spectrometry coupled with liquid chromatography (LC/MS). BBB integrity was estimated by the ratio of atenolol brain to plasma concentration (C_brain_ and C_plasma_, respectively). This ratio is described by the partition coefficient (Kp).

$$ Kp=\frac{C_{brain}}{C_{plasma}} $$

The P-gp and BCRP transport activities were estimated by means of the digoxin and prazosin Kp, respectively.

### LC/MS assay for digoxin, prazosin and atenolol quantification

Brains were mixed in ultrapure water (2 mL/g of tissue) using an Ultraturrax T65 system (IKA-Werke, Staufen, Germany). Extract suspensions (400 μL) were submitted to protein precipitation with 1 mL of methanol previously spiked with internal standard (digoxin-d3 and atenolol-d7 4 μg/mL). After centrifugation (20000 g; 15 min; 4 °C) the supernatant was dried under nitrogen at 40 °C. The dried extracts were resuspended in 1 mL 0.75 M NH_4_OH / methanol (80:20 *v/v*). Plasma (150 μL) was diluted with 150 μL of 0.75 M NH_4_OH / methanol (80:20 *v/v*) previously spiked with internal standard. Both brain and plasma extracts were submitted to solid-liquid extraction on isolute SLE+ columns 1 or 6 mL (Biotage). The two eluates (3 mL of dichloromethane/isopropanol (70:30 *v/v*) then 3 mL of dichloromethane/isopropanol (70:30 *v/v*) + 0.2 % formic acid) were pooled and evaporated to dryness. The dry extracts were resuspend in 200 μL of 5 mM ammonium acetate/methanol (95:5 *v/v*). Chromatography was performed using a Shimadzu HPLC system LC 20 AD on a Kinetex C18 column (Phenomenex). The total run time was 5 min and the flow rate was 0.4 mL/min. Analyte (20 μL) was injected onto the column placed in an oven at 40 °C.

Detection was done by tandem mass spectrometry (Finnigan TSQ Quantum Discovery with Xcalibur and LC Quan softwares, Thermo) in positive electrospray mode. Tuning parameters were: capillary voltage 3 kV, source temperature 200 °C. The multiple reaction monitoring transitions for analytes were as follows: m/z digoxin 798.5 > 651.4, m/z prazosin 384.19 > 247.14, m/z atenolol 267.18 > 145.1. Analytes were quantified by means of calibration curves using digoxin-d3 or atenolol-d7 as internal standard. For plasma and brain extract assay, calibration ranges were from 1.0 to 200 ng/mL.

### Isolation of brain microvasculature endothelial cells

Rat brain capillaries were isolated as described previously [26, 55]. 6 rats from three relevant time groups (5 min; 2 h and 28 days) were used. Brains were extracted and stored in Hanks balanced salt solution (HBSS) supplemented with 1 % (*v/v*) PSN on ice. The remainder of the isolation took place under aseptic conditions. The brains were cut sagittally into two halves and the cerebral cortices emptied of white matter. The meninges and the associated vessels were cleaned off by rolling on Whatman 3 mm chromatography paper. The homogenized tissue was pelleted by centrifugation at 1500 rpm for 5 min. The pellet containing the microvessels was digested in HBSS-1 % PSN solution, 1 mg/mL collagenase/dispase, 10 U/μL DNase-I, and 1 μg/mL TLCK for 1 h at 37 °C. Digested tissue was then pelleted by centrifugation at 1500 rpm for 5 min at 4 °C. To remove myelin, the pellet was resuspended in 20 % (*w/v*) BSA in HBSS-1 % PSN solution and centrifuged at 2800 rpm for 30 min. The resulting floating white matter corresponding to the brain parenchyma fraction and centrifugation medium were removed carefully. The microvessel pellets were washed then frozen at −80 °C. The remaining parenchyma fraction was resuspended in centrifugation medium then half-dissolved in HBSS-1 % PSN solution and pelleted by centrifugation at 1500 rpm for 15 min. The brain parenchyma pellet was then washed several times before being frozen at −80 °C.

### Cell culture

Rat primary BECs and glial cells were isolated and cultured as previously described [26]. Briefly, for BECs, brain tissues were digested enzymatically (1 g/L collagenase/dispase, 20 U/mL DNAse I, 0.147 mg/L TCLK in HBSS, 1 h at 37 °C). A 20 % BSA gradient was used for isolation of capillaries. After a second enzymatic digestion, cells were plated in 75 cm^2^ coated culture flasks in EBM medium completed by the EGM-2 MV SingleQuots kit (Lonza, Basel, Switzerland). Cultures were maintained at 37 °C in a humidified 5 % CO_2_ atmosphere for 5–6 days before being trypsinized and frozen.

### BBB cell-based model

For BBB modeling, glial cells were seeded at a density of 5700 cells/cm^2^ on transwell plates in a glial-specific basal medium as previously reported [26]. BECs were plated on the upper side of a coated polyester transwell membrane (pore size 0.4 mm, Costar, Dutscher sa, Brumath, France) at a density of 71400 cells/cm^2^ in a BEC-specific medium. Microplates were then incubated at 37 °C in a humidified 5 % CO_2_ atmosphere for 5 to 7 days before treatment. Upper and lower chambers will be referred to as apical and basal compartments, respectively, throughout this manuscript. 4 sera from controls and 4 sera from treated animals were tested. Each collected serum from controls and treated animals were diluted at 1/16 in the BEC medium. BECs were exposed to diluted rat serum in the apical compartment for 24 h. Transport experiments were performed in six wells for each serum.

### Transport experiments

After exposure to sera, exposure medium were removed then transwells with BECs were transferred to new plates. 0.5 and 1.5 mL of transfer buffer (150 mM NaCl, 5.2 mM KCl, 2.2 mM CaCl_2_, 0.2 mM MgCl_2_, 6 mM NaHCO_3_, 2.8 mM glucose and 5 mM Hepes) was added to the apical and basolateral compartments or donor and acceptor chambers, respectively. Lucifer yellow (LY) was added to the donor chamber to a final concentration of 0.1 mM and, after 30 min, aliquots from acceptor and donor chambers were collected for determination of tracer concentration by fluorescence counting. The apparent permeability (P_app_) value for LY was calculated as follows:

$$ {\mathrm{P}}_{\mathrm{app}} = \mathrm{d}\mathrm{Q} = \mathrm{d}\mathrm{T} \times \mathrm{A} \times {\mathrm{C}}_0 $$

where dQ/dT is the amount of LY transported per time-point, A is the membrane surface area and C_0_ the initial donor concentration. The mass balance (R) was calculated as:

$$ \mathrm{R}\left(\%\right) = 100 \times \left[\left(\mathrm{A} + \mathrm{D}\right)/{\mathrm{D}}_0\right] $$

where A and D are the amounts of LY in the acceptor and donor chambers and D_0_ is the amount introduced at t: 0. Mass balances of LY were between 80 and 120 %. As LY permeability values on the order of 10^−6^ cm/s were obtained previously in various *in vitro* BBB models [33, 56–58] , we considered that beyond 5.10^−6^ cm/s the monolayer is disrupted.

### Transcription profiling

RNA was isolated from rat BECs, brain parenchyma fraction or cells isolated from the BBB *in vitro* model using the RNeasy plus universal tissue minikit or the RNeasy Mini kit, respectively (Qiagen, France), according to the manufacturer’s instructions. The concentration and purity of the RNA samples were assessed spectrophotometrically at 260 and 280 nm using the NanoDrop ND-1000 instrument (NanoDrop Technologies, Wilmington, DE, USA). The A260/280 ratio ranged between 1.8 and 2.

A sample of 0.5 μg of total RNA was converted to cDNA with random primers in a total of 10 μL using an RT2 first stand kit (Qiagen, France). The cDNA was diluted with DNA/RNAse-free distilled water to a volume of 110 μL.

The quantitative expression of various cytokines, chemokines, transporters and structural proteins was determined using 0.4 μM cDNA for each primer set in the RT2 Pathway Focus profiler array from Qiagen. The RT2 Profiler array consists of a previously validated qRT-PCR primer set (1 μL) for relevant cytokines, chemokines: Il-1β, CXCL1 and IP-10; tight junction proteins: Cldn5 and Ocln; ABC transporters: *Abcb1* and *Abcg2* and housekeeping genes (Hprt, GAPDH). Thermocycling was carried out in a CFX96 real-time PCR detection system (Bio-Rad) using SYBR green fluorescence detection. The final reaction mixture contained 2 μL of diluted cDNA, 1 μL of one of the specific primer, 12.5 μL of distilled water and 9.5 μL of SYBR green master mix. The specific amplification conditions were 2 min at 50 °C, 10 min at 95 °C followed by 40 amplification cycles at 95 °C for 0.5 min, and 60 °C for 1 min to reinitialize the cycle again. The specificity of each reaction was also assessed by melting curve analysis to ensure the presence of only one product. Relative gene expression values were calculated as 2^−ΔCT^, where ΔCT is the difference between the amplification curve (CT) values for genes of interest and housekeeping genes (hypoxanthine guanine phosphoryltransferase or Hprt; glyceraldehyde phosphodehydrogenase or GAPDH). If the CT was higher than 35, we considered the expression level too low to be applicable.

### Immunofluorescence

Brain sections embedded in OCT and cut on a cryostat (10 μm) were prepared for either occludin or claudin-5, and von Willebrand factor (vWF) double immunofluorescence, or IL-1β. Brain sections were air-dried for 30 min and fixed in ice-cold acetone for 30 min and then rinsed in PBS. Sections were then incubated with 3 % BSA for 60 min at RT, rinsed in PBS, and incubated with 150 μL per section of the appropriate primary antibody (occludin: 1:1000, Abcam, Cambridge, MA; clauin-5: 1:100, Invitrogen/Life Technologies, Carlbad, CA) and an FITC-tagged vWF (1:1000 dilution, Abcam) or IL-1β (1:1000, Abcam) alone; diluted in wash buffer [1 part 5 % blocking solution (0.5 mL normal rabbit serum in 10 mL 3 % *w/v* bovine serum albumin) and 4 parts phosphate-buffered saline (PBS)] for 1 h at room temperature (RT) and then rinsed 3 times with PBS. The slides were incubated in 150 μL per section of the appropriate secondary antibody with either Alexa Fluor 555 or Alexa Fluor 546 (1:1000 dilution, Vector Laboratories, Biovalley, Marne la Vallée, France) in the dark for 1 h at RT. Slides were rinsed 3 times in PBS and subsequently incubated with Hoechst nuclear stain (1 μL/mL; 150 μL/section) for one minute, rinsed again then coverslipped with Aqueous Gel Mount (TBS, Fisher Scientific, Waltham, MA). Slides were imaged by fluorescence microscopy at 10x and 40x, using the appropriate excitation/emission filters, digitally recorded, and analyzed by image densitometry using Image J software (NIH). A minimum of 3 locations on each section (2 sections per slide), 3 slides and n = 3 per group were processed/analyzed. Only vessels >50 μm were used for analysis.

### Statistical Analysis

Analyses were performed using the Prism 5.1 program (GraphPad Software, Inc, San Diego CA). Statistical comparisons conducted herein were accomplished using variance analysis (two-way ANOVA) two determinants being time and treatment for organs Ti distribution followed by the Bonferroni post test. mRNA expression of cytokines and chemokines were analyzed using one tailed Mann-Whitney test and two tailed Man Whitney test for other mRNA expressions (tight junctions proteins and transporters). Immunofluorescence end points were analyzed using one-way ANOVA between treatment groups followed by the Holm-Sidak post hoc test (SigmaPlot SyStat Software Inc, San Joes, CA) and data were expressed as mean ± SD. Changes were considered statistically significant at *P* < 0.05.

## Additional files

Additional file 1:
**BBB integrity assessment after IV injection of 10 mg/kg TiO**_**2**_ **NPs in rats.** BBB integrity was estimated by the ratio between atenolol concentrations in brain and plasma (partition coefficient or Kp). Each data point represents the mean ± SD of n = 8 rats. Statistical comparison was performed by two tailed Mann-Whitney test, * *P* < 0.05. (TIFF 4945 kb) Additional file 2:
**Effect of 24 h TiO**_**2**_ **NPs exposure on BEC epithelium integrity.** TiO_2_ NPs concentrations from 0 to 100 μg/mL were applied to the apical pole of the *in vitro* BBB model for 24 h. Data are apparent permeability coefficient of sucrose. Each data point represents the mean ± SD of at least 2 experiments. The horizontal dotted line represents the usual limit value of 8.3 10^-6^ cm.s^−1^ beyond which the BEC monolayer is considered to be disrupted. The blue zone represents sucrose apparent permeability coefficient for the controls (Mean ± SD). (TIFF 61 kb)

## Abbreviations

TiO_2_NPs: Titanium dioxide nanoparticles
CNS: Central nervous system
IV: Intravenous
IL-1β: Interleukin-1β
CXCL1: Chemokine Ligand 1
IP-10: Gamma inductible protein-10
BECs: Brain endothelial cells
P-gp: P-glycoprotein
BCRP: Breast cancer resistance protein
TEM: Transmission electron microscopy
SEM: Scanning electron microscopy
EDX: Energy dispersive X ray
BET: Brunauer-Emmett-Teller
DLS: Dynamic light scattering
ICP-MS: Inductively couple plasma mass spectrometry
LOQ: Limit of quantification
Kp: Partition coefficient
P_app_: Apparent permeability
LY: Lucifer yellow
GFAP: Glial fibrillary acid protein
RT: Room temperature
TNF-α: Tumor Necrosis Factor - α
TNF-R1: Tumor necrosis factor receptor 1
