# Supplementary figures and images for: Tissue biodistribution of intravenously administrated titanium dioxide nanoparticles revealed blood-brain barrier clearance and brain inflammation in rat
Source: Part Fibre Toxicol. 2015 Sep 4;12:27. doi: 10.1186/s12989-015-0102-8 (PMC4559366; doi:10.1186/s12989-015-0102-8)

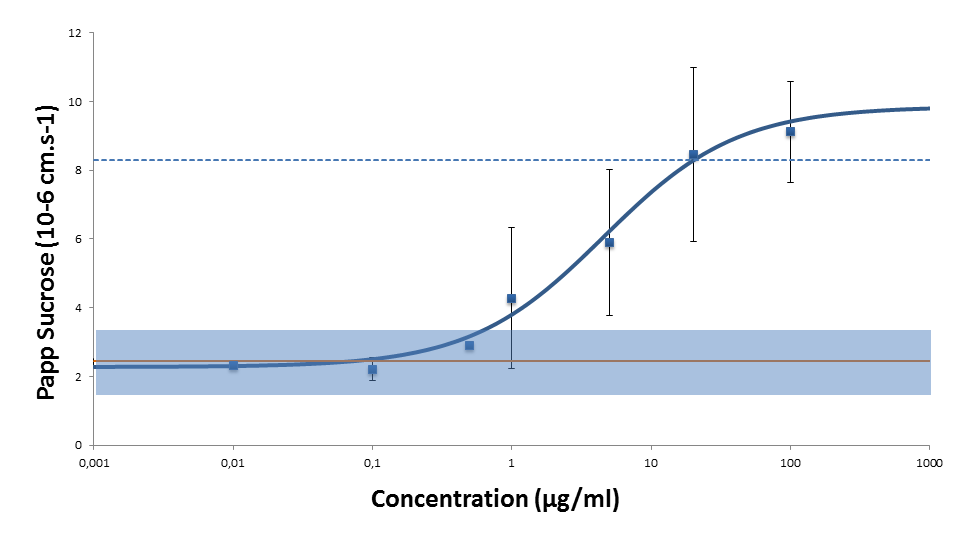

Supplement: Additional file 2: — Effect of 24 h TiO 2 NPs exposure on BEC epithelium integrity. TiO2 NPs concentrations from 0 to 100 μg/mL were applied to the apical pole of the in vitro BBB model for 24 h. Data are apparent permeability coefficient of sucrose. Each data point represents the mean ± SD of at least 2 experiments. The horizontal dotted line represents the usual limit value of 8.3 10-6 cm.s−1 beyond which the BEC monolayer is considered to be disrupted. The blue zone represents sucrose apparent permeability coefficient for the controls (Mean ± SD). (TIFF 61 kb) [file 12989_2015_102_MOESM2_ESM.tif]
